# Mangiferin from *Pueraria tuberosa* reduces inflammation *via* inactivation of NLRP3 inflammasome

**DOI:** 10.1038/srep42683

**Published:** 2017-02-20

**Authors:** Ramakrishna K. Bulugonda, Kotha Anil kumar, D. Gangappa, Harshavardhan Beeda, Gundala Harold Philip, Dowlathabad Muralidhara Rao, Syed M. Faisal

**Affiliations:** 1 Department of Biotechnology, Sri Krishnadevaraya University, Anantapur, India; 2 National Institute of Animal Biotechnology (NIAB), Hyderabad, India; 3 School of Life Sciences, University of Hyderabad, Hyderabad, India

## Abstract

Recent reports have demonstrated the role of phyto-constituents in modulating inflammatory responses. Mangiferin isolated from *Mangifera indica* is known to induce potent anti-oxidative, anti-diabetic and anti-inflammatory activity. However, the molecular mechanism of its anti-inflammatory activity is not properly understood. In this study we have isolated Mangiferin from the tubers of *Pueraria tuberosa* (PT-Mangiferin) and analysed the mechanism of its potent anti-inflammatory effects in LPS stimulated RAW 264.7 mouse macrophage cell line and in a carrageenan induced air pouch model. PT-Mangiferin was non-toxic to primary cells but showed significant toxicity and apoptotic effect on cancerous cells. It significantly reduced the production of pro-inflammatory mediators (COX-2, iNOS and TNF-α) in LPS stimulated RAW 264.7 cells. Further, it has also reduced the generation of ROS and inhibited LPS induced NF-kB translocation in these cells. Additionally, PT-Mangiferin significantly reduced inflammation in a mouse air pouch model by inhibiting the infiltration of monocytes and neutrophils and reducing the production of cytokines. These effects were mediated via inactivation of NLRP3 inflammasome complex and its downstream signalling molecules. Taken together these results suggest that PT-Mangiferin is potent anti-inflammatory compound that reduces inflammation and holds promise in development of herbal based anti-inflammatory therapeutics in future.

Inflammation is a protective response after infection or injury. It is a biological process which eliminates the inciting stimulus, promotes tissue repair/wound healing and establishes memory so that host shows faster and specific response upon future encounter. Inflammation is associated with many disorders such as atherosclerosis, thrombosis, arthritis, asthma, neurodegenerative disorders and even cancer^1^.

NLRs (nucleotide binding domain, leucine rich repeat containing receptors) are associated with inflammation and their dysregulated activation is responsible to several inflammatory diseases, metabolic disorders and auto-inflammatory immune disorders. NLRs upon activation form multi protein complex called ‘inflammasome’ whose activation results in conversion of pro-caspase-1 to caspase-1 which results in conversion of several substrates like pro IL-1β, IL-18 to their active forms and mediate activation of NF-κB and MAPK signaling pathways^2^,^3^. One of the most intensively studied inflammasomes is the NLRP3 inflammasome that contains NLRP3 sensor, ASC adaptor and caspase-1 protease. Upon stimulation, NLRP3 inflammasome components assemble into large cytoplasmic complexes and activation of caspase-1 eventually leads to the maturation and secretion of IL-1β^4^. The NLRP3 inflammasome is an important contributor to various inflammatory diseases^5^. The rapidly growing evidence for excessive inflammasome activation in a variety of common diseases highlights the need for discovery of potentially therapeutic inhibitors of NLRP3 inflammasome.

The conventional anti-inflammatory drugs used to relieve inflammatory processes are either too expensive or come with several side effects. In this sense, pharmaceutical companies have supported the search and development of drugs with anti-inflammatory activity from plants. Natural products have long been used in medicine and drugs based on these products have been also used in the prevention and treatment of many inflammatory disorders. They interfere with inflammatory mechanisms preventing prolonged inflammatory processes that are problematic for human health. Several natural products were known to have anti-inflammatory effects but very few products like Aloe Vera, Curcumin, Ginseng and Resveratrol have demonstrated their anti-inflammatory effects via inactivation of NLRP3 inflammosome^6^,^7^,^8^,^9^,^10^,^11^,^12^,^13^.

Mangiferin is a natural polyphenol with a C-glycosylxanthone structure. The primary source of Mangiferin is the mango tree (*Mangifera indica*); however, it is also present in other medicinal herbs like Iris unguicularis and in the honey bush, a popular herbal tea from South Africa^14^. In addition, Magiferin is also found in *Anemarrhena asphodeloides* Bunge, which has been widely used in Chinese traditional medicine system for the treatment of diabetes^15^. Mangiferin is known for its analgesic, antidiabetic, antisclerotic, antibacterial and antiviral activities^15^,^16^,^17^. Few studies have demonstrated the anti-inflammatory property of Mangiferin, however, the molecular mechanism of its effect on inflammaosomes has not been completely elucidated^18^,^19^,^20^.

In this study we isolated Mangiferin from the tubers of *Pueraria tuberosa* (PT-Mangiferin) and determined its anti-inflammatory mechanism in most reliable mouse air pouch model of inflammation.

## Results

### PT-Mangiferin is non-toxic to primary cells but exerts toxic and anti-proliferative effect on cancer cell lines

**Figure 1:**
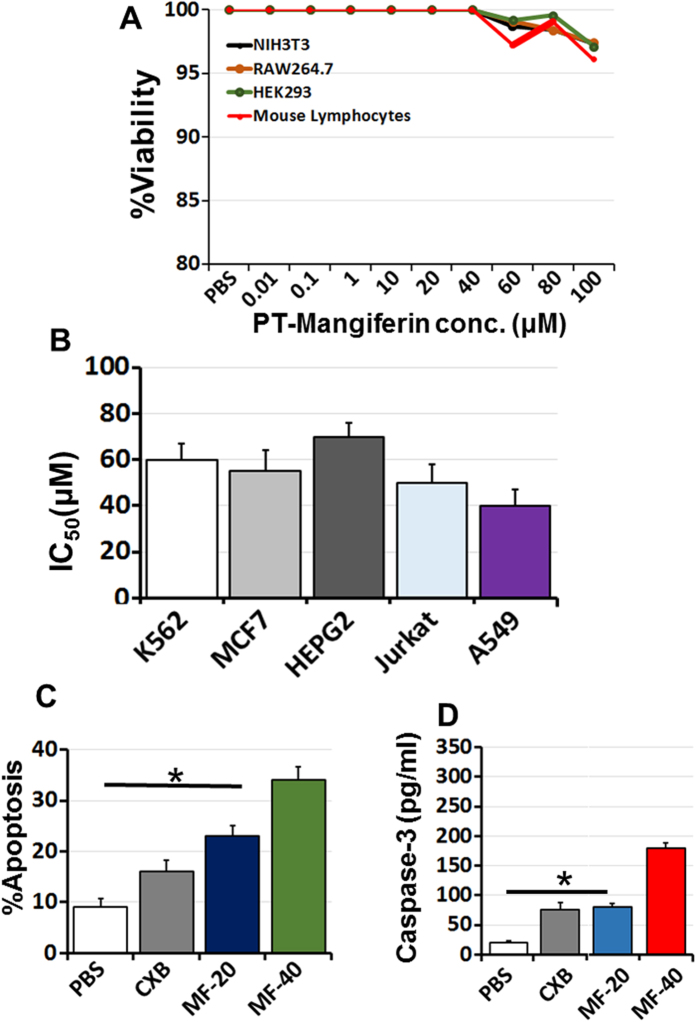
PT-Mangiferin is nontoxic to primary cells but exerts anti-proliferative effect via inducing apoptosis in cancer cells. (**A**) Various primary cells and cell lines (murine fibroblast NIH-3T3, RAW264.7, HEK293 and mouse lymphocytes) were incubated with or without PT-Mangiferin (0.01–100 μM) for 48 h and then the cell viability was measured by MTT assay as described in materials and methods. (**B**) Various cancer cell lines (K562, MCF7, HEPG2, Jurkat E6.1 cells and A549) were incubated with or without PT-Mangiferin (0.01–100 μM) for 48 h and then the cell viability as measured by MTT assay as described in materials and methods. The data represents concentration of PT-Mangiferin inducing toxicity in 50% cells (IC_50_). (**C**) Apoptosis induced by PT-Mangiferin (20, 40 μM) in A549 cells at 72 h was determined by Annexin V binding using flow cytometry and represented as percent apoptotic cells. (**C**) Caspase-3 levels in A549 cells were detected by ELISA using specific antibody. Data are mean ± SEM and representative of three independent experiments. *P < 0.05. PBS- Phosphate buffer saline, LPS-Lipopolysaccharide (1 μg/ml), CXB-Celecoxib (20 μM), MF-20- PT-Mangiferin (20 μM), MF-40- PT-Mangiferin (40 μM).

PT-Mangiferin didn’t show any significant level of toxicity to normal cell lines or primary cells (mouse fibroblast NIH-3T3, RAW264.7, HEK293 and mouse lymphocytes) even at concentration of 100 μM as determined by MTT assay (Fig. 1A). However, it showed significant level of cytotoxicity to various cancer cell lines like K562 (IC_50_ 60 μM), MCF7 (IC_50_ 56 μM), HEPG_2_ (IC_50_ 69 μM), Jurkat cells (IC_50_ 48 μM) and A549 (IC_50_ 40 μM) at different inhibitory concentrations (Fig. 1B). PT-Mangiferin induced maximum anti-proliferative effect in A549 cells and also induced apoptosis in these cells in dose dependent manner (Fig. 1C). This data correlated with expression of active caspase-3 in A549 cells as there was significant increase (p < 0.05) in its level with increasing concentration of PT-Mangiferin (Fig. 1D).

### PT-Mangiferin significantly reduced the generation of ROS and inhibited the LPS induced NF-κB translocation in mouse macrophages

**Figure 2:**
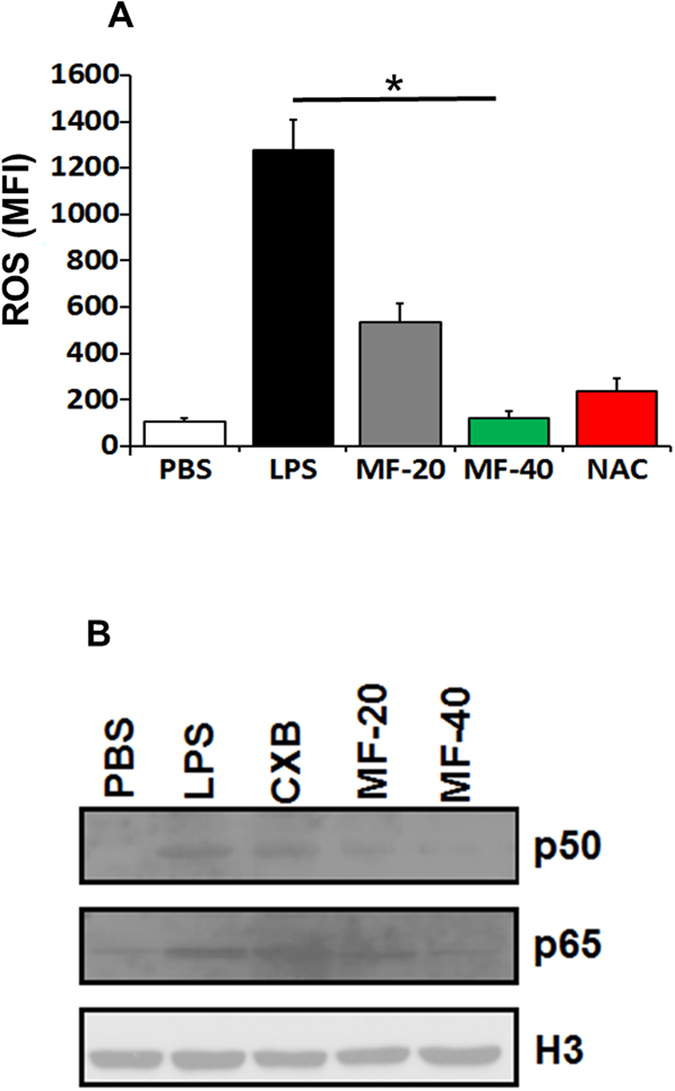
PT-Mangiferin reduced generation of ROS and inhibited translocation of NF-κB in RAW 264.7 cells. (**A**) RAW 264.7 cells were treated with PBS, LPS (1 μg/ml) followed by treatment with Celecoxib (20 μM) or different doses of PT-Mangiferin (20, 40 μM) and cells were kept for 16 h at 37 °C/5% CO_2_. ROS levels were measured by Flow cytometry. N-Acetyl Cysteine, a ROS inhibitor was used as a positive control. (**B**) Nuclear extract of the cells with above treatments were prepared and analyzed for translocation of p50 and p65 sub units using specific antibodies as described in materials and methods. Histone 3(H3) was used as internal control. The gels were run under same experimental conditions. PBS- Phosphate buffer saline, LPS-Lipopolysaccharide (1 μg/ml), CXB- Celecoxib (20 μM), MF-20- PT-Mangiferin (20 μM), MF-40- PT-Mangiferin (40 μM).

The anti-inflammatory effect of PT-Mangiferin was evaluated by examining its effect on generation of Reactive Oxygen Species (ROS). PT-Mangiferin significantly reduced the LPS induced generation of ROS (p < 0.05) in dose dependent manner (Fig. 2A). PT-Mangiferin at 40 μM dose was more effective in reducing the level of ROS than standard ROS inhibitor, N-acetyl cysteine (NAC). NF-κB translocation is responsible for expression of inflammatory proteins like COX-2, iNOS, TNF-α. However, pre-incubation of cells with PT-Mangiferin significantly inhibited LPS induced NF-κB translocation in dose dependent manner as significantly reduced levels of p50 and p65 (p < 0.05) were observed in nuclei of cells (Fig. 2B).

### PT-Mangiferin reduced the production of LPS induced pro-inflammatory mediators by mouse macrophages

**Figure 3:**
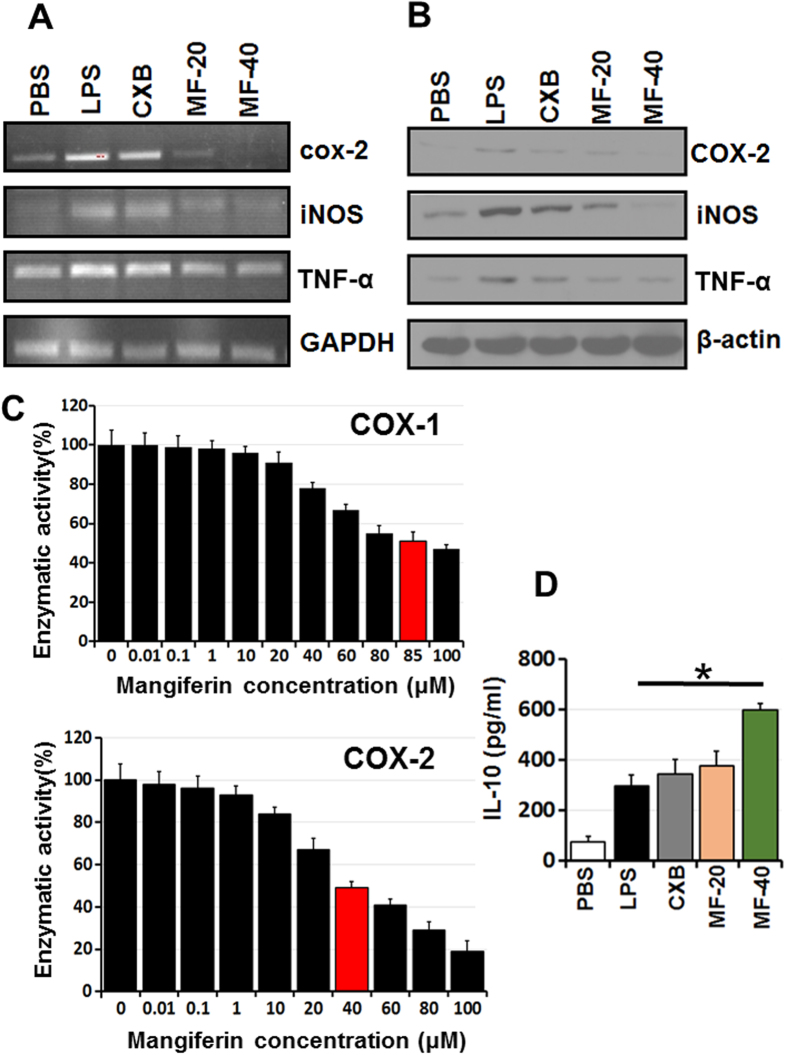
PT-Mangiferin reduced the production of proinflammatory mediators in RAW 264.7 cells. (**A**) RAW 264.7 cells were treated with PBS, LPS (1 μg/ml) and LPS followed by treatment with Celecoxib (20 μM) or different doses of PT-Mangiferin (20, 40 μM). The cells were kept for 16 h at 37 °C/5% CO_2_. Semi-quantitative PCR was performed for analyzing expression of cox-2, iNOS and TNF-α at transcription level. GAPDH was used as internal control. (**B**) Cytoplasmic extract of the cells with above treatment were run on SDS-PAGE and then transferred to nitrocellulose membrane followed by probing with specific antibodies for analyzing expression of COX-2, iNOS and TNF-α at protein level. β-actin was used as internal positive control. (**C**) RAW 264.7 cells were treated with different concentration of PT-Mangiferin (0.01–100 μM) and enzymatic activity of COX-1 and COX-2 was determined as described in materials and methods. The data is represented as percent enzyme activity (**D**) Analysis of IL-10 by ELISA in culture supernatants of RAW264.7 cells. Data are mean ± SEM and representative of three independent experiments. *P < 0.05. PBS- Phosphate buffer saline, LPS-Lipopolysaccharide (1 μg/ml), CXB- Celecoxib (20 μM), MF-20- PT-Mangiferin (20 μM), MF-40- PT-Mangiferin (40 μM).

PT-Mangiferin significantly reduced the expression of inflammatory mediators (COX-2, iNOS, and TNF-α) induced by LPS in RAW 264.7 cells (p < 0.05). The reduced expression was observed both at gene expression level (Fig. 3A) which correlated with protein levels (Fig. 3B). PT-Mangiferin has shown to be more effective than standard non-steroidal anti-inflammatory drug (NSAID), Celecoxib (CXB) at similar dose (20 μM) in reducing the levels of COX-2 and iNOS (Fig. 3). PT-Mangiferin inhibited both COX-1 and COX-2 as revealed by their reduced enzymatic activities with IC_50_ value of 85 μM and 40 μM respectively (Fig. 3C). PT-Mangiferin also induced production of significant levels of anti-inflammatory cytokine (IL-10) in RAW 264.7 cells (Fig. 3D). These results indicate that PT-Mangiferin is potent anti-inflammatory compound.

### PT-Mangiferin significantly reduced the carrageenan induced inflammation in a mouse air pouch model

**Figure 4:**
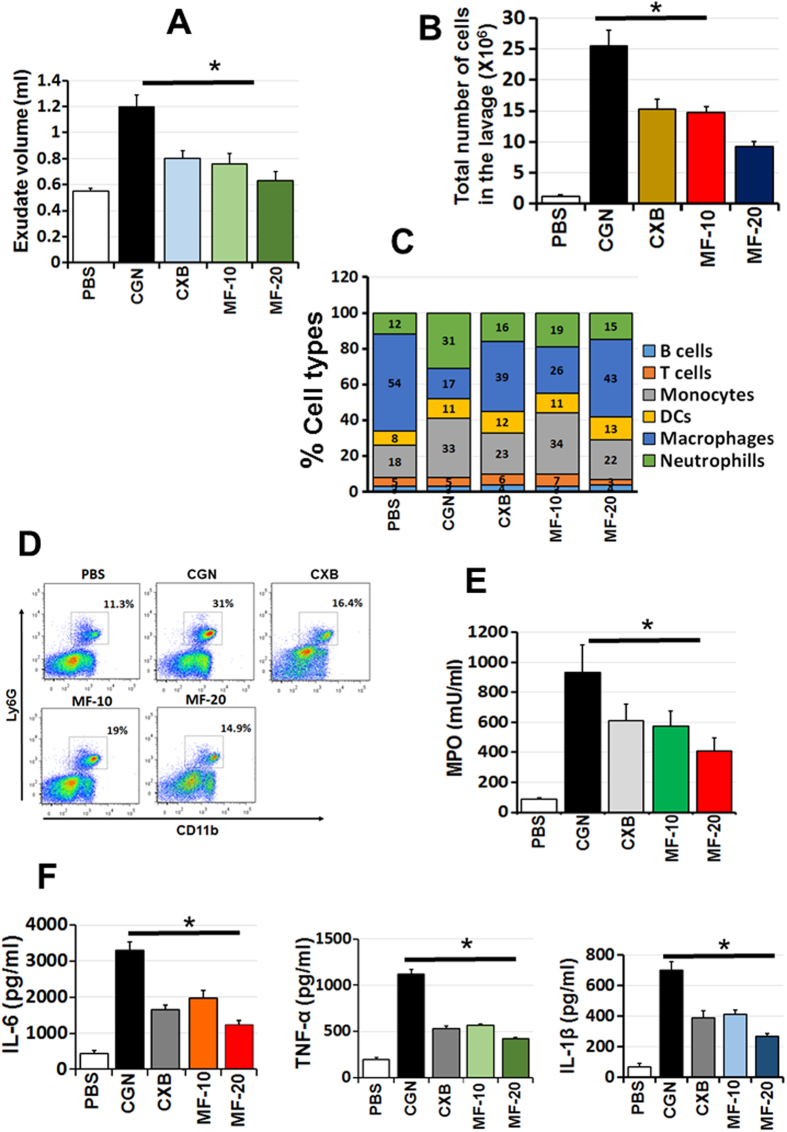
PT-Mangiferin reduced the infiltration of monocytes and neutrophils and production of pro-inflammatory cytokines in the air pouch. Animals from different treatment groups were killed, air pouch were exposed and exudates were recovered with 500 μl PBS as described in materials and methods. (**A**) Volume of exudate recovered from each group. (**B**) Total cells in the exudates from each group as determined by counting. (**C**) Percent of each cell type in the exudates as analyzed by Flow cytometry. (**D**) FACS plots showing neutrophil population in each group. (**E**) MPO analysis. (**F**) Analysis of cytokines (IL-6, TNF-α, IL-1β) in the inflammatory exudates by ELISA. PBS- Phosphate buffer saline, CGN-Carrageenan (1.5%, 0.5 ml), CXB- Celecoxib (10 mg/kg body weight), MF-10- PT-Mangiferin at 10 mg/kg body weight, MF-20- PT-Mangiferin at 20 mg/kg body weight.

**Figure 5:**
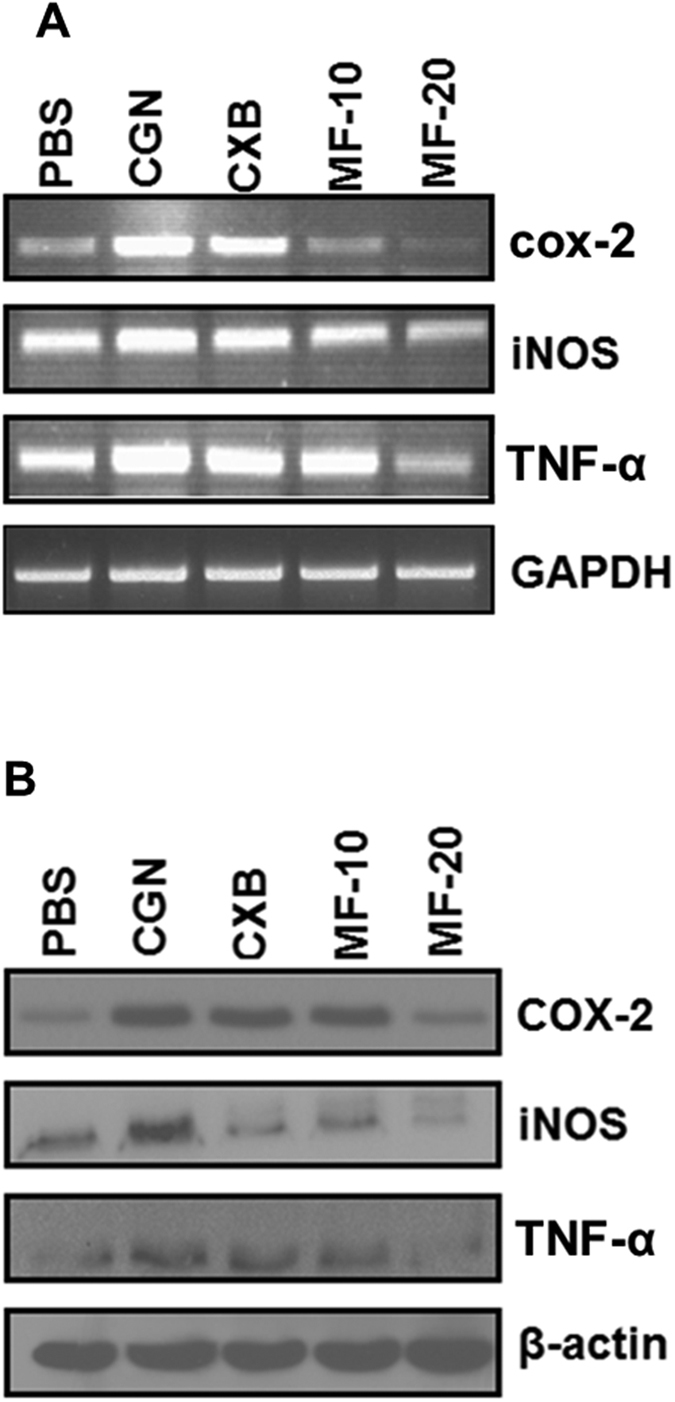
PT-Mangiferin significantly reduced the production of proinflammatory mediators in the mouse air pouch tissues. (**A**) cDNA obtained from air pouch tissues with different treatments as described in material and methods were analyzed for expression of cox-2, iNOS and TNF-α at transcription level. GAPDH was used as positive control. (**B**) Proteins obtained from air pouch tissue was subjected to Immunoblot for analyzing expression of COX-2, iNOS and TNF-α. β-actin was used as internal positive control. Data are mean ± SEM and representative of thee independent experiments. *P < 0.05. PBS- Phosphate buffer saline, CGN- Carrageenan (1.5%, 0.5 ml), CXB-Celecoxib (10 mg/kg body weight), MF-10- PT-Mangiferin at 10 mg/kg body weight, MF-20- PT-Mangiferin at 20 mg/kg body weight.

**Figure 6:**
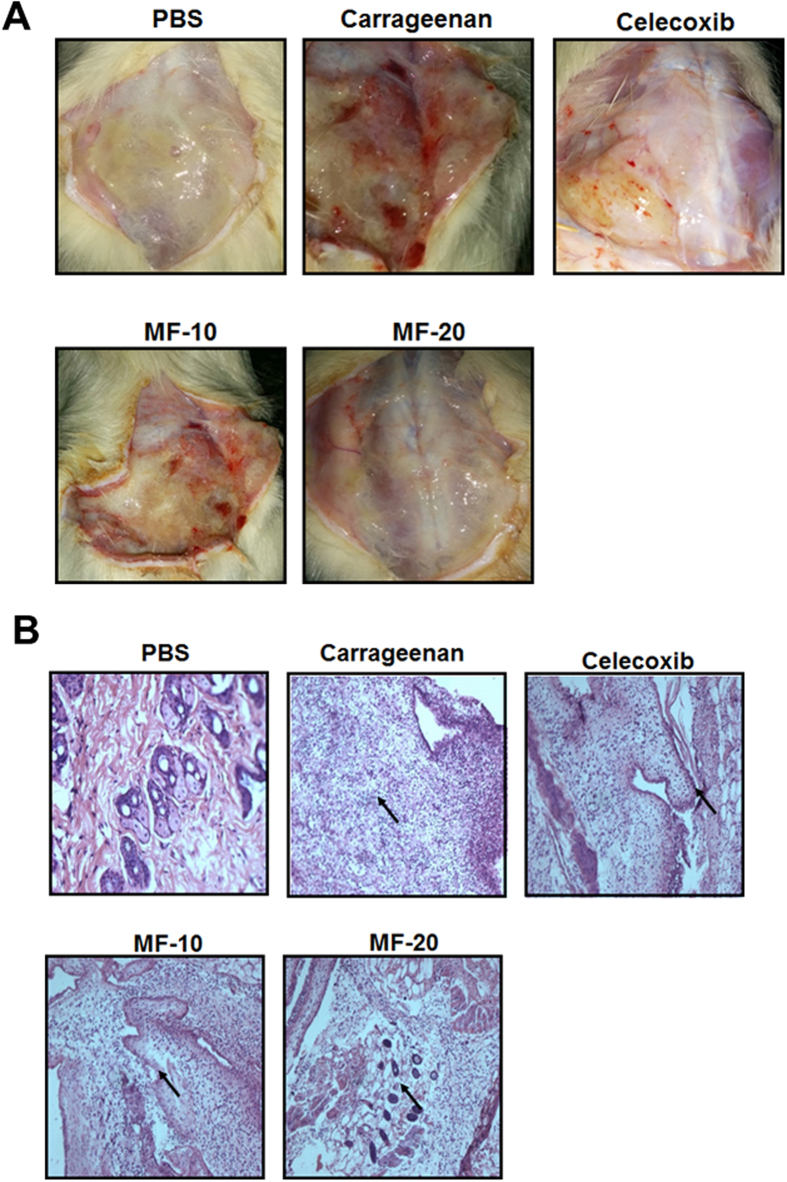
PT-Mangiferin significantly reduced the carrageenan induced inflammation in a mouse air pouch model. (**A**) Pictures of mouse air pouch tissue from various groups taken 24 h after treatment. (**B**) Photomicrographs showing the histological sections of pouch tissue 24 h after administration. The data is representative of two independent experiments. PBS- Phosphate buffer saline, CGN- Carrageenan (1.5%, 0.5 ml), CXB- Celecoxib (10 mg/kg body weight), MF-10- PT-Mangiferin at 10 mg/kg body weight, MF-20- PT-Mangiferin at 20 mg/kg body weight.

The air pouch exudates of the mice treated with carrageenan and PT-Mangiferin were analyzed after 24 h for total number of cells, phenotypes and cytokines. PT-Mangiferin treatment significantly reduced the volume and infiltrating cells in the air pouch cavity (Fig. 4A,B). Carrageenan induced infiltration of large number of polymorphonuclear cells (PMNs) predominantly monocytes and neutrophils which were significantly reduced after treatment with PT-Mangiferin. Cells within the inflammatory exudate identified by flow cytometry were B cells, T cells, Monocytes, Dendritic Cells and Neutrophils. B and T cells were present in low numbers (Fig. 4C). There was significant reduction in the level of neutrophils (p < 0.05) after PT-Mangiferin treatment in dose dependent manner as determined by FACS which correlated to reduced MPO activity in the exudate (Fig. 4D,E). The low number of cells in the PT-Mangiferin treated animals also correlated to significantly reduced production of pro-inflammatory cytokines (p < 0.05) like IL-6, TNF-α and IL-1β in the exudate (Fig. 4F). PT-Mangiferin also significantly reduced the expression of inflammatory mediators (COX-2, iNOS, and TNF-α) as analyzed in air pouch tissue (p < 0.05). Reduced expression was observed at the gene expression level (Fig. 5A) which correlated with protein levels (Fig. 5B). The classical symptoms of acute inflammation - redness, swelling and infiltration of cells were clearly observed in the air pouch lining of carrageenan treated animal (Fig. 6A). The inflammatory reaction gradually progressed with time and reached a peak at 24 h after carrageenan treatment. The carrageenan treated pouch tissue showed increase in the size of blood vessels which were significantly reduced in mice treated with PT-Mangiferin (10 mg/kg body weight) and were almost completely abrogated in mice treated with higher dose (Fig. 6A). Histopathological analysis clearly showed that in PT-Mangiferin treated animals the inflammatory reaction and cell infiltration was less when compared to animals treated with carrageenan alone (Fig. 6B). PT-Mangiferin at a dose of 20 mg/kg body weight was more effective as tissue histology results were close to normal or PBS control.

### PT-Mangiferin reduces inflammation by inactivating NLRP3 inflammasome

**Figure 7:**
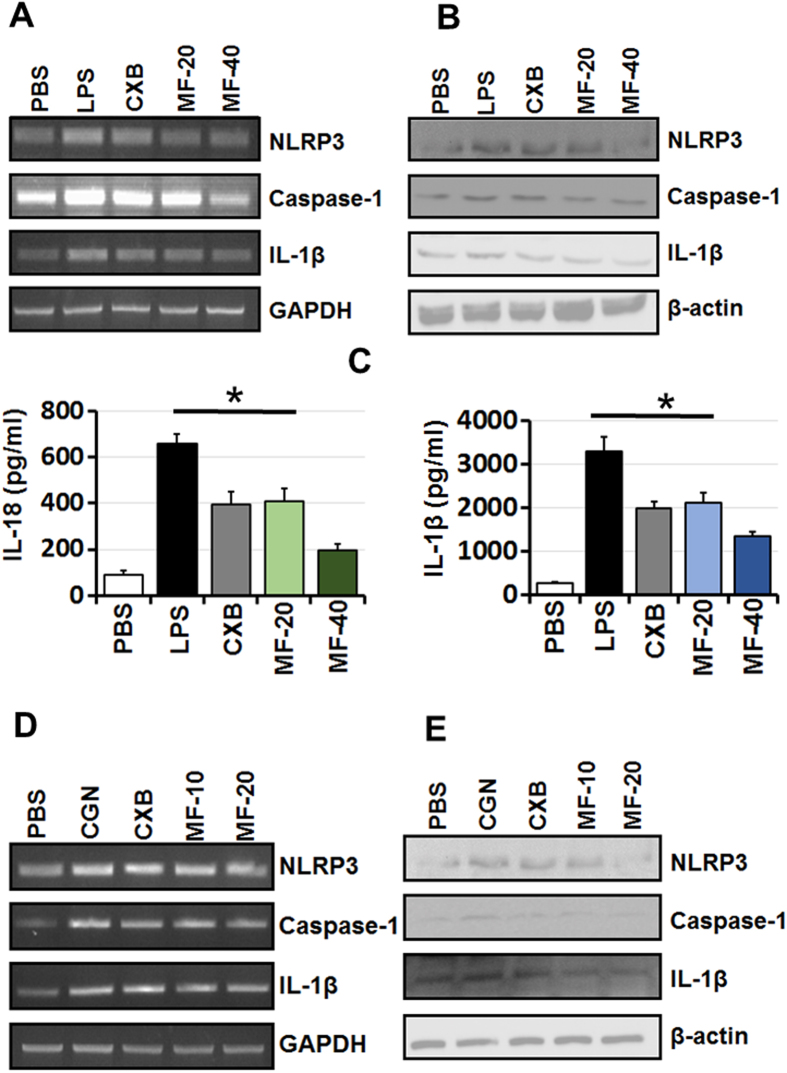
PT-Mangiferin reduces inflammation by inactivating NLRP3 inflammasome. (**A**) cDNA obtained from RAW 264.7 cells with different treatments as described in material and methods were analyzed for expression of NLRP3, Caspase-1 and IL-1β at transcription level. GAPDH was used as positive control. (**B**) Analysis of expression of NLRP3, Caspase-1 and IL-1β in RAW 264.7 cells at protein level. β-actin was used as internal positive control. (**C**) Analysis of IL-1β and IL-18 by ELISA in the culture supernatants of RAW264.7 cells. (**D**) cDNA obtained from air pouch tissues with different treatments were analyzed for expression of NLRP3, Caspase-1 and IL-1β α at transcription level as described in material and methods. GAPDH was used as positive control. (**E**) Analysis of expression of NLRP3, Caspase-1 and IL-1β from air pouch tissues at protein level. β-actin was used as internal positive control. Data are mean ± SEM and representative of three independent experiments. *P < 0.05 PBS- Phosphate buffer saline, LPS-Lipopolysaccharide (1 μg/ml), CXB- Celecoxib (20 μM), MF-20- PT-Mangiferin (20 μM), MF-40- PT-Mangiferin (40 μM), CGN- Carrageenan (1.5%, 0.5 ml), CXB-Celecoxib (10 mg/kg body weight), MF-10- PT-Mangiferin at 10 mg/kg body weight, MF-20- PT-Mangiferin at 20 mg/kg body weight.

PT-Mangiferin significantly inhibited various components involved in inflammosome activation in LPS induced macrophages (RAW 264.7) both at gene expression and protein level (p < 0.05). There was significant reduction in transcript and protein levels of NLRP3, Caspase-1 and IL-1β in cells treated with PT-Mangiferin (Fig. 7A,B). The significant reduced levels (p < 0.05) of IL-1β and IL-18 cytokines further supported this observation (Fig. 7C). PT-Mangiferin also significantly reduced the expression of NLRP3, Caspase-1 and IL-1β in carrageenan treated air pouch tissue both at gene expression and protein level. These results indicate that PT-Mangiferin reduces inflammation by inactivating or inhibiting the NLRP3 inflammasome.

## Discussion

The currently available anti-inflammatory agents mainly consisting of NSAIDs, glucocorticoids, immunosuppressant drugs and biologicals are either not effective enough or associated with intolerable side effects^21^. Thus, the discovery of new anti-inflammatory compounds is currently in great demand. The NLRP3 inflammasome is an important contributor to diverse inflammatory diseases such as Alzheimer, atherosclerosis, diabetes etc. Few recent studies have discovered some promising candidates, however, there are currently no clinically available inhibitors of NLRP3^22^,^23^. The extracts of many plants are known to act as anti-inflammatory agents however mechanism of action is largely unknown^24^,^25^ Mangiferin isolated from *Mangifera indica* has shown to mediate anti-oxidant, antidiabetic and anti-inflammatory property mainly by targeting MAP kinases and NF-κB signalling pathways^26^,^27^,^28^,^29^,^30^,^31^. Recent studies have shown that Mangiferin inhibits NLRP3 inflammasome in liver and endothelial cells to protect against liver injury and endothelial dysfunction^32^,^33^. In this study we have exploited the mouse air pouch model which is most reliable model for inflammation and confirmed that anti-inflammatory activity of Mangiferin isolated from *P. tuberosa* (PT-Mangiferin) is indeed associated with its ability to inhibit NLRP3 inflammosomes.

In the current study, PT-Mangiferin effectively reduced inflammation in dose dependent manner and provided protection against the acute inflammatory response induced by carrageenan in mice. Although we used three doses of PT-Mangiferin both *in vitro* and *in vivo*, the lowest dose (10 μM or 5 mg/kg body weight) had no significant effect in reducing the inflammation (data not shown). On testing the effect of PT-Mangiferin on various normal cell lines and primary cells (mouse fibroblast, macrophages and spleenocytes) our results showed that it did not cause any significant level of toxicity upto 100 μM concentration but caused anti-proliferative effect in various cancer cells with IC_50_ value ranging from 40 μM to 70 μM. It showed maximum toxicity to A549 lung cancer cell line by inducing significant level of apoptosis in these cells (Fig. 1C,D). Our data is in accordance to recent report of Mangiferin showing anti-proliferative effect on various cancer cell lines with maximum effect on A549 cells^34^. PT-Mangiferin was found to be more effective in reducing the generation of ROS in RAW 264.7 cells than standard inhibitor NAC (Fig. 2A). The xanthonoid structure with C-glycosyl linkage and polyhydroxy component of PT-Mangiferin is believed to be crucial for its ability to scavenge ROS. The reduced generation of ROS correlated to inhibition of NF-κB signaling as there was reduced levels of p50 and p65 in nuclei (Fig. 2B). NF-κB p50/p65 heterodimers in most cells are held in the cytoplasm by an inhibitor molecule, IκBα. The specific stimulatory signals such as LPS, PMA or TNF-α can cause degradation of IκBα and release of p50/p65 causing transport of transcriptionally active heterodimers to the nucleus leading to induction of transcription of target genes^35^. ROS interacts with NF-κB signaling pathways in many ways. The transcription of NF-κB dependent genes influences the levels of ROS in the cell, and in turn, the levels of NF-κB activity are also regulated by the levels of ROS. Depending on the context, ROS can both activate and inhibit NF-κB signaling. For instance ROS often stimulates the NF-κB pathway in the cytoplasm, but inhibits NF-κB activity in the nucleus^35^. Since our data shows that PT-Mangiferin inhibited production of COX-2 enzyme it is likely that NF-κB is regulating the production of ROS though COX-2 (Fig. 3A,B).

Further, PT-Mangiferin inhibited the LPS induced production of proinflammatory mediators like COX-2, iNOS, TNF-α by RAW 264.7 cells (Fig. 3). Cyclooxygenase exist in two isoforms, COX-1 and COX-2 where latter is rapidly induced during inflammation and contributes to acute inflammation though production of proinflammatory signaling molecules^36^,^37^. Although COX-2 appears to be the dominant source of prostaglandin formation in inflammation, the role of COX-1 in contributing to the acute inflammatory response cannot be ruled out. COX-1 is constitutively expressed in resident inflammatory cells and there is evidence for induction of COX-1 during LPS-mediated inflammatory response and cellular differentiation^38^. Consistent to our previous study, our data shows that PT-Mangiferin is inhibiting both COX-1 and COX-2 (Fig. 3C)^39^. However we have used much lower dose where it is predominantly targeting COX-2. In contrast to standard NSAIDs, it seems that anti-inflammatory mechanism of PT-Mangiferin is via targeting both COX-1 and COX-2. Nitric oxide (NO) is another important pro-inflammatory mediator released during the inflammatory process. It is produced by the inducible nitric oxide synthase (iNOS) during inflammation. This mediator can induce oxidative stress in macrophages and also modulate T cell development and cytokine production^40^. In our study, PT-Mangiferin treatment caused a significant reduction in levels of pro-inflammatory mediators including iNOS and TNF-α. The reduction in these mediators may be due to inhibition of NF-κB signaling as iNOS (NOS2) is heavily upregulated by NF-κB^35^.

Several studies have described the use of the mouse air pouch model as a very consistent and straightforward model to investigate inflammatory response, creating an ideal environment for collection and phenotypic analysis of cells migrating into the pouch space^41^,^42^. In this study, we exploited this model to evaluate the effect of PT-Mangiferin on carrageenan induced infiltration of inflammatory cells, mediators and cytokines. PT-Mangiferin significantly reduced the exudate volume, infiltration of monocytes and neutrophils, pro-inflammatory cytokines (IL-6, TNF-α and IL-1β) and MPO in the air pouch of carrageenan treated animals (Fig. 4). This effect was dose dependent and level of neutrophils correlated to MPO activity which is an enzyme present in primary granules and is considered to be an important marker for inflammatory response in a variety of acute and chronic inflammatory conditions^43^. Since NO acts on the endothelium increasing the vascular permeability and consequently increasing exudation, it seems that the reduced level of pro-inflammatory mediator like iNOS in PT-Mangiferin treated animals may have contributed to reduced generation of NO leading to reduction in exudate volume^44^. The reduction in cell number achieved by treatment of PT-Mangiferin seems mainly due to the inhibition of neutrophil migration and reduction in inflammatory mediators. These results indicate that PT-Mangiferin showed an anti-inflammatory property by inhibiting migration of leukocytes (mainly monocytes and neutrophils) and reducing pro-inflammatory enzymes and cytokines in the exudates (Figs 4 and 5). The type of infiltrating cells and their numbers present in inflammatory responses generally varies with time from induction with phagocytes being predominant during the early phases of inflammation, whereas lymphocytes become more evident in chronic inflammation^45^. Since we have not analyzed the lavage post 24 h it was not possible to determine if PT-Mangiferin induced the migration of lymphocytes in the pouch cavity. Consistent with the flow cytometry analysis of exudate cells, at 24 h histology data also showed that PT-Mangiferin treated pouches appear less inflamed, with decreased cellularity (lower number of infiltrating cells) similar to PBS-treated pouches (Fig. 6).

To investigate whether the anti-inflammatory mechanism of PT-Mangiferin occurs *via* inactivation of NLRP3 inflammasome, we analyzed the expression of NLRP3, caspase-1 and IL-1β in LPS stimulated macrophages and carrageenan induced inflamed air pouch tissue. Our results showed that PT-Mangiferin significantly inhibited expression of NLRP3, caspase-1 and IL-1β in both macrophages and air pouch tissue. The reduced production of these mediators correlated to levels of IL-18 and IL-1β in mouse macrophages (Fig. 7). It is likely that PT-Mangiferin is inhibiting the expression of Caspase-1 and IL-1β by reducing the generation of ROS as it has been shown that mitochondrial ROS plays an important role in NLRP3 inflammasome activation^46^,^47^,^48^.

In conclusion, we have uncovered the fact that anti-inflammatory mechanism of PT-Mangiferin is via inhibiting NF-κB signaling, COX-1, COX-2 and inactivation of NLRP3 inflammaosomes highlighting its ability to modulate several points in the inflammatory pathway. The major advantage of using PT-Mangiferin as anti-inflammatory compound is that it may confer greater protective effects than compounds that have single targets and can also be effective at lower doses. Therapeutically, our data indicates that PT-Mangiferin has potent anti-inflammatory property and has potential to be repurposed as drug against various inflammatory diseases.

## Materials and Methods

### Chemicals and Reagents

MTT (3-(4, 5-dimethylthiazol-2-yl)-2,5-diphenyl tetrazolium bromide), Tris, Ethylene diamine tetra acetic acid (EDTA), Trypsin-EDTA, Di-ethyl dithio-carbamate (DDC), Tween-20, Hematin, Glycerol, Phenol, Ammonium sulphate, Carrageenan, COX-1 and COX-2 enzymes were purchased from Sigma-Aldrich (St. Louis, MO, USA). Celecoxib was a generous gift from Unichem Laboratories (Mumbai, India). The stock solutions of PT-Mangiferin (100 mg/ml) was prepared in dimethyl sulphoxide and further dilutions were made in PBS for treatment in *vitro* and *in vivo*. Polyclonal antibodies to, COX-2, IL-1β and TNF-α were purchased from Santa Cruz Biotechnology (California, U.S.A.). iNOS antibody was from Thermo scientific. All other chemicals and solvents were of analytical grade and purchased from authorized standard companies.

### Cell culture

Human Leukemic cell line (K562), Hepatocellular carcinoma cells (HepG2), Breast carcinoma cells (MCF-7), Human T cell leukemic cells (Jurkat E6.1), Human lung carcinoma cells (A549), Mouse fibroblast (NIH-3T3), Human Embryonic Kidney cells (HEK293) and mouse macrophage cell line (RAW 264.7) were obtained from NCCS (Pune, India). The cells were grown in their recommended mediums either in Dulbecco’s Modified Eagle Medium (DMEM) or RPMI1640 (GIBCO, USA) supplemented with 2 mM glutamine, 10% heat-inactivated FBS, 100IU/ml penicillin and 100 μg/ml streptomycin in a humidified atmosphere with 5% CO_2_ at 37 °C.

### Isolation and purification of Mangiferin from *Pueraria tuberosa*

Mangiferin was isolated from the methanolic extract of tubers of *P. tuberosa* (here referred to as PT-Mangiferin) as described previously^39^. Briefly, the tubers of *P. tuberosa* were collected from Tirumala hills, Andhra Pradesh, South India. A voucher specimen (DG-11) has been deposited in the herbarium of the Department of Botany, Sri Venkateswara University, Tirupati. Air-dried and powdered (3 kg) tubers of *P. tuberosa was* successively extracted with n-hexane (3 × 5 L), Ethyl acetate (EtOAc, 3 × 5 L) and Methyl alcohol (MeOH, 3 × 5 L). The MeOH extract was concentrated under reduced pressure to yield a brown colored viscous residue. The MeOH extract on purification over a silica gel column using n-hexane and n-hexane-EtOAc step gradient afforded 3 compounds and one among them was PT-Mangiferin. The structures of compounds 1–3 were elucidated by various NMR techniques including ^1^H, ^1^H COSY, HSQC, HMBC, NOESY experiments and ESI-TOF Mass Spectrometry. PT-Mangiferin was dissolved in DMSO and prepared as stock of 100 mM.

### Cell proliferation assay

Various cell lines or primary cell (mouse fibroblast NIH-3T3, RAW264.7, HEK293 and mouse lymphocytes) and various cancer cell lines (K562, MCF7, HEPG2, Jurkat E6.1 cells and A549) were seeded in 96 well plates in recommended medium at a density of 5 × 10^4^ cells/well. The cells were treated with different concentrations of PT-Mangiferin (0.01 μM to 100 μM) for 48 h and cell proliferation was assessed colorimetrically by standard MTT assay described by Mossman^49^. The effect of PT-Mangiferin on growth inhibition was assessed as percent cell viability; control cells treated with the PBS were considered 100% viable. Supernatant from 0.1% Triton X-100 treated cells (100% lysis) was used as positive control.

### Apoptosis assay

Apoptosis induced by PT-Mangiferin on A549 cells was measured using flow cytometry (to quantify the levels of detectable phosphatidylserine on the outer membranes of apoptotic cells) and caspase-3 activity. A549 cells were seeded in 6 well cultures plate. At 60–70% confluency, cells were incubated with different concentrations of PT-Mangiferin (10, 20 and 40 μM) and Celecoxib (20 μM) for 72 h. Cells were stained with FITC labelled Annexin V using Apoptosis Detection Kit (Cat#556547, BD, USA) according to manufacturer’s protocol and then analyzed by FACS. For determining the activated caspase-3 protein levels, the cells were lysed and total protein was quantified with the BCA Protein Assay Reagent Kit. The active caspase-3 in the lysate was detected by ELISA by using mouse cleaved caspase-3 antibody (Cat#DYC835, R&D Systems).

### Measurement of ROS

RAW 264.7 cells were seeded in 6 well plates at a density of 2 × 10^5^ cells/well and then pre incubated with different concentrations of PT-Mangiferin (10, 20 and 40 μM). The cells were stimulated with LPS (1 μg/ml) for 16 h and then treated with 10 μM DCFH-DA (2′,7′-dichlorodihydrofluorescein diacetate) and further incubated for 15 min at 37 °C. The cells were collected, washed with PBS and total of 10000 events were analyzed in FL1 channel by Flow cytometry (FACS caliber, BD, USA).

### Animals

Adult Balb/c male mice weighing 20–25 g were used in the present study. They were fed with a standard chow pellet diet, had free access to water, and were maintained on a 12:12-h light-dark cycles. All procedures in this study were approved by the Animal Ethical Committee of National Institute of Animal Biotechnology, Hyderabad and Sri Krishnadevaraya University, Anantapur. All the methods were carried out in accordance with the approved protocols and relevant guidelines.

### Air pouch model of inflammation

Carrageenan treated mice air pouch model of inflammation was developed as described previously^10^. Air cavities were produced by sub-cutaneous injections of 5 ml of sterile air into the intra-capsular area on the dorsal side of the animal. An additional 3 ml of air was injected into the cavity every three days. Seven days after the initial air injection, 0.5 ml of 1.5% (w/v) solution of carrageenan dissolved in saline was injected directly into the pouch to produce an inflammatory response. Control animals received 0.5 ml of saline only. PT-Mangiferin and Celecoxib were injected thee hours prior to injection of carrageenan into the pouch cavity. Animals were divided into 5 different groups as follows: PBS treated; carrageenan (0.5 ml of 1.5% (w/v) carrageenan in saline) treated; carrageenan + Celecoxib (10 mg/Kg body weight); carrageenan + PT-Mangiferin (5 mg/Kg body weight) treated; carrageenan + PT-Mangiferin (10 mg/Kg body weight) treated; carrageenan + PT-Mangiferin (20 mg/Kg body weight) treated. For the time course studies, animals were sacrificed by cervical dislocation at various time points after the injection. Pouch tissue was carefully dissected and inflammatory exudates were recovered by lavaging the pouches with 500 μl of sterile PBS. The pouch lining was separated from the muscle and dissected out, and rinsed in saline before processing further. The tissue was used to study the expression of inflammatory genes at RNA and protein level and lavage/exudate was used to analyze infiltrating cells and cytokines. For checking toxicity of PT-Mangiferin, splenocytes were isolated from normal untreated animals using standard procedure.

### Preparation of cytoplasmic and nuclear extracts

RAW 264.7 cells were cultured in 6-well plates (4 × 10^6 ^cells/well) with or without LPS (1 μg/ml), Celecoxib (20 μM) and in the presence or absence of different concentration of PT-Mangiferin (10, 20, 40 μM). After culture the cells were collected and washed twice with cold PBS, lysed in 400 μL of cold buffer A (10 mM HEPES pH 7.9, 10 mM KCl, 1 mM EDTA, 1 mM phenylmethanesulphonylfluoride (PMSF), 1 mM EGTA, 1 mM dithiotheitol (DTT1mg/ml Aprotinin, 1 mg/ml Leupeptin and 1 mg/ml Pepstatin A). After 15-min incubation on ice, 0.1% NP-40 was added to the homogenates and the tubes were vigorously rocked for 1 min. Then the homogenates were centrifuged (20,800 g × *g*, 5 min) at 4 °C. The supernatant fluid (cytoplasmic extracts) was collected and stored in aliquots at −70 °C. The nuclear pellets were washed once with cold buffer A, then suspended in 50 μL of cold buffer B (20 mM HEPES, pH 7.9, 420 mM NaCl, 0.1 mM EDTA, 1 mM PMSF, 1 mM DTT, 1 mg/ml Aprotinin, 1 mg/ml Leupeptin and 1 mg/ml Pepstatin A) and vigorously rocked at maximum speed at 4 °C for 30 min. The solution was clarified by centrifugation at 20,800× *g* for 5 min, and the supernatant fluid (nuclear extract) was stored in aliquots at −70 °C. The protein concentration was determined according to the Bradford method. Air pouch tissue homogenate was prepared by homogenizing in 100 mM Tris-HCl (pH 8.0) buffer containing 0.3 M Mannitol, 1 mM EGTA, 1 mM EDTA, 4 mM K_2_HPO_4_, 1 mM DTT, 1 mM Sodium orthovanadate, 0.1% SDS, 2 mM PMSF and 40 μl/ml complete protease inhibitor. The homogenate was centrifuged for 30 min at 10,000 rpm at 4 °C and the protein content in the supernatant was measured by Bradford method^9^.

### Western Blotting

Equal amounts of protein from RAW 264.7 cells and tissue homogenates were separated by SDS-PAGE and then transferred electrophoretically to nitrocellulose membranes (Millipore, Bedford, MA). After blocking with 5% nonfat milk powder in PBS (pH 7.4, 0.05% (v/v) Tween-20), the membranes were incubated overnight at 4 °C with corresponding primary Abs, for the detection of COX-2, iNOS, Caspase-1, NLRP3, TNF-α, IL-1β and β-actin according to the manufacturer’s instructions. After washing with PBS, the membranes were incubated for 1–2 h at 37 °C with the appropriate HP-conjugated anti-rabbit IgG secondary Ab (1:5000, CST, USA). The peroxidase-positive bands were detected using an ECL detection kit (Bio-Rad Cat#1705060).

### Enzymatic assay

Enzymatic activities of both COX-1 and COX-2 were measured according to the method of Copeland *et al*. with slight modifications, using a chomogenic assay based on the oxidation of N,N,N′,N′-tetramethyl-p-phenylenediamine (TMPD) during the reduction of PGG2 to PGH2^50^,^51^,^52^.

### Cytokine analysis

Cytokine sandwich ELISA kits (R&D systems) were used to measure cytokine levels, following the manufacturer’s instructions. IL-10, IL-18 and IL-1β were measured from culture supernatant of RAW264.7 cells. IL-6, TNF-α and IL-1β were measured from exudates obtained from various groups.

### Flow cytometric analysis of inflammatory exudate

The cells in the exudate were counted on an automatic cell counter (Bio-Rad) using trypan blue to assess viability. The cells (1 × 10^6^) were suspended in FACS buffer (PBS, 2% (v/v) fetal calf serum, and 0.05% (w/v) NaN3), blocked with Fc block (anti-CD16/32) and then stained with lineage specific and fluorochrome labelled monoclonal antibodies against T cells (CD3-FITC), B cells (B220-PE), Monocytes (CD11b-APC), Dendritic cells (CD11c-PerCP), Macrophages (F4/80-PE-Cy7) and Neutrophils (Ly6G-APC-Cy7). The cells were washed with FACS buffer and then 50,000 cells were acquired in Flow cytometer (FACS caliber, BD, USA). The data was analyzed by FLOW JO software (Tree star Inc.).

### Myeloperoxidase assay

The Myeloperoxidase (MPO) activity was evaluated in the exudates obtained from various groups by using MPO colorimetric assay kit (Cat# MAK068-1KT Sigma, USA) following manufacturer’s instructions. Briefly, 100 μL of lavage fluid was mixed with 400 μL (4 volume) of MPO Assay Buffer and homogenized with sonication for 10 seconds and three subsequent freeze and thaw cycles. The sample was then centrifuged at 13,000 g for 15 min at 4 °C. The supernatant was then used for the measurement of MPO activity using kit according to manufacturer’s protocol. The results were expressed in milli units per milliliter.

### Histopathology of air pouch tissue

Air pouch tissues from control and experimental animals were rinsed in PBS and fixed in Bouin’s fixative (70% saturated picric acid, 25% formaldehyde and 5% glacial acetic acid) overnight followed by thorough washing with distilled water. Tissues were then dehydrated sequentially in 70%, 80%, 90% alcohol and finally in absolute alcohol for 10 min each. After dehydration, the tissue was processed in alcohol and benzene (3:1 for 10 min, 1:1 for 10 min, benzene and paraffin (1:1) for 10 min) to embed in paraffin wax. The tissue was placed in molten paraffin for 3 h to allow infiltration of paraffin into the tissue and then allowed to harden. Thin sections (10 μm) were taken on Leitz microtome and mounted on polylysine-coated slides. Sections were deparaffinised by incubating in xylene for 10 min, rehydrated by sequential incubations in 90%, 80% and 70% alcohol for 10 min each. The tissue sections were observed under light microscope at 400x magnification and photographs were taken.

### Reverse Transcriptase Polymerase Chain Reaction (RT-PCR)

RAW 264.7 cells (5 × 10^6^ cells) were seeded in 90 mm Petri plates. Cells were pre incubated with different concentrations of PT-Mangiferin (10, 20 and 40 μM) or celecoxib (20 μM) for 3 h and then stimulated with LPS (1 μg/ml) for 24 h. Cells were recovered and suspended in Trizol. Similarly pouch tissues obtained from different groups were homogenized in Trizol reagent. Total cellular RNA was extracted using RNAeasy kit (Qiagen) according to the manufacturer’s instructions. RNA quality was checked by running on a Formaldehyde gel for 16 S and 18 S RNA bands and also on Bio-analyzer. The RNA quantity was assessed by UV spectroscopy and purity by 260/280 ratio. cDNA of each sample was prepared by using 2 μg of RNA, 1 μl M-MLV reverse transcriptase, 1 mM dNTP and 1 μl oligo dT according to manufacturer’s standardized protocol (Promega Corporation, WI, USA). RT-PCR was performed in 20 μl reaction containing 50 ng cDNA, 2 μl 10X reaction buffer, 1U Taq DNA polymerase, 0.2 mM dNTP, 100 pmol of forward and reverse primer (Table 1- Supp. info) to detect cox-2, iNOS, TNF-α, NLRP3, Caspase-1, IL-1β, and GAPDH. After amplification, PCR products were electrophoresed on 1% agarose gels and visualized by ethidium bromide staining on UV irradiation (BIO-RAD, Universal hood II).

### Statistical analysis

Statistical analysis was performed by multiple software’s like Excel, Analysis of Variance (ANOVA) and Bonferroni Post hoc test for multiple comparisons wherever applicable. *P-*value was determined by the Student’s T-test. *P-*value of less than 0.05 was considered as a significant difference.

## Additional Information

**How to cite this article**: Bulugonda, R. K. *et al*. Mangiferin from *Pueraria tuberosa* reduces inflammation *via* inactivation of NLRP3 inflammasome. *Sci. Rep.* **7**, 42683; doi: 10.1038/srep42683 (2017).

**Publisher's note:** Springer Nature remains neutral with regard to jurisdictional claims in published maps and institutional affiliations.

## Supplementary Material

Supplementary Information
