# Supplementary material for: Mangiferin from Pueraria tuberosa reduces inflammation via inactivation of NLRP3 inflammasome
Source: Sci Rep. 2017 Feb 20;7:42683. doi: 10.1038/srep42683 (PMC5316935; doi:10.1038/srep42683)
Supplement: Supplementary Information [file srep42683-s1.pdf]

Supplementary Information

Mangiferin from *Pueraria tuberosa* reduces inflammation *via* inactivation of NLRP3 inflammasome

Ramakrishna. K. Bulugonda[1†], Kotha Anil kumar[2,3†], Gangappa D[3], Harshavardhan Beeda[1], Gundala Harold Philip[1], Muralidara Rao[1*], Syed M. Faisal[2*]

[1]Department of Biotechnology, Sri Krishnadevaraya University, Anantapur, India
[2]National Institute of Animal Biotechnology (NIAB), Hyderabad, India
[3]School of Life Sciences, University of Hyderabad, Hyderabad, India

Corresponding authors

Muralidhara Rao, SKU, Anantapur (muralidararao@gmail.com) or Syed M Faisal, NIAB, Hyderabad (faisal@niab.org.in)

The supplementary file contains Table 1.

Table1. Primers used for RT-PCR

S.NO	Gene	Primer Sequence
**1**	GAPDH	F- AGGTCATCCCAGAGCTGAACG<br>R- CACCCTGTTGCTGTAGCCGTA
**2**	IL-1β	F- GCAACTGTTCCTGAACTCAACT<br>R- ATCTTTTGGGGTCCGTCAACT
**3**	TNF-α	F- AGCACAGAAAGCATGATCCG<br>R- CTGATGAGAGGGAGGCCATT
**4**	cox-2	F- GAAGTCTTTGGTCTGGTGCCTG<br>R- GTCTGCTGGTTTGGAATAGTTGC
**5**	iNOS	F- TCCTACACCACACCAAAC<br>R- CTCCAATCTCTGCCTATCC
**6**	NLRP3	F- CTTCCAGACTGGTGAACTGCTG<br>R- TCTCCAAGGGCATTGCTTCG
**7**	Caspase-1	F- GAGCTTCAATCAGCTCCATCAG<br>R- AATGTCCCGGGAAGAGGTAGA
